# Inhibitory Effect of *Astragalus* Polysaccharide on Premetastatic Niche of Lung Cancer through the S1PR1-STAT3 Signaling Pathway

**DOI:** 10.1155/2023/4010797

**Published:** 2023-01-20

**Authors:** Ming Shen, Yan-jun Wang, Zhao-hua Liu, Yan-wen Chen, Qian-kun Liang, Yang Li, Hai-xia Ming

**Affiliations:** ^1^School of Basic Medicine, Gansu University of Chinese Medicine, Lanzhou, China; ^2^Basic Discipline of Integrative Traditional Chinese and Western Medicine, Gansu University of Chinese Medicine, Lanzhou, China; ^3^Gansu University Key Laboratory for Molecular Medicine & Chinese Medicine Prevention and Treatment of Major Diseases, Gansu University of Chinese Medicine, Lanzhou, China; ^4^Key Laboratory of Chronic Diseases for Prevention and Treatment of Traditional Chinese Medicine, Lanzhou, China; ^5^Affiliated Hospital of Gansu University of Chinese Medicine, Lanzhou, China; ^6^Research Center of Traditional Chinese Medicine, Gansu Province, Lanzhou, China

## Abstract

As a common malignant tumor, the morbidity and mortality of lung cancer have been rising in recent years. The concept of “premetastatic niche” may lead to a revolutionary change in antitumor metastasis therapeutic strategies. Traditional Chinese medicine with multitargets and lower poisonous agents may be a potentially effective means to intervene in the “premetastatic niche (PMN)” to prevent and treat tumor metastasis. *Astragalus* polysaccharide (APS) is a substance with strong immune activity in *Astragalus membranaceus* that has excellent biological activities such as immunomodulation, anti-inflammatory, and antitumor. In this study, we constructed a tumor lung metastasis animal model to explore the intervention mechanism of APS on the premetastatic niche. We found that APS inhibited the formation of the lung premetastatic niche and inhibited the recruitment of myeloid-derived suppressor cells (MDSCs) in the lung. Mechanistically, we showed that the proteins and gene expression of S1PR1, STAT3, and p-STAT3 in the S1PR1/STAT3 signaling pathway were suppressed by APS. In line with the above findings, our results confirmed that APS may inhibit the accumulation of MDSCs in the premetastatic niche through the intervention of the S1PR1-STAT3 signaling pathway to achieve the antitumor effect.

## 1. Introduction

Lung cancer is the most common malignant tumor in the respiratory system [1]. According to the latest cancer data statistics of the International Agency for Research on Cancer (IARC), the number of new cases of lung cancer reaches 2.2 million and 1.8 million deaths in 2020. In terms of mortality, it ranks first among all cancer types. Tumor metastasis to distant organs is the cause of death in most cancer patients [2]. Therefore, the development of new treatment modalities for lung cancer is urgently required, and a deeper understanding of the biology of the biological process of lung metastasis will help establish new therapeutics aimed at preventing and treating cancer. Although surgical treatment, immunotherapy, and targeted therapies have been applied clinically, tumor metastasis and postoperative recurrence rates remain high [3]. The concept of the premetastatic niche (PMN) is considered a key factor in metastasis. Formation of PMN promotes tumor cell invasion and escape as well as tumor cell proliferation in secondary organs [4]. Therefore, uncovering the molecular mechanisms of PMN formation will help to elucidate the mechanisms of tumor metastasis and find new targets for intervention.

Clinical studies have proved that myeloid-derived suppressor cells (MDSCs) are valuable predictors in the development of lung cancer. They expand and mediate immunosuppression during tumor progression, and their activation secretes cytokines, chemokines, and enzymes that promote tumor cell invasion, proliferation, adhesion, and chemotaxis and ultimately lead to tumor invasion and metastasis [5]. Therefore, intervention in MDSCs may provide new targets for anticancer therapy. The signal transducer and activator of transcription (STAT) proteins are a family of cytoplasmic transcription factors that share the structure and organize functional modular domains [6]. As an important member of the STAT transcription factor family, STAT3 is an important transcription factor that mediates intercellular signal transduction [7]. Studies have shown that the sustained activation of STAT3 in T cells and myeloid cells at primary tumor sites promotes tumor angiogenesis, immunosuppression, tumor survival, and metastasis [8]. Because of the role of the STAT3 signaling pathway in tumor formation and metastasis, most scholars regard it as a potential therapeutic target for cancer therapy. Sphingosine-1-phosphate (S1P) is an important bioactive sphingolipid metabolite, and sphingosine kinase is a key rate-limiting enzyme for S1P synthesis, classified as SphK1 and 2, which regulates a variety of physiological and pathological processes [9, 10]. As a bioactive lipid mediator, S1P can mediate signal transduction by combining with the cell surface receptor S1PR1, which has been proven to enhance the proliferation, survival, and metastasis of tumor cells [11]. It is worth mentioning that S1PR1 plays an indispensable role in maintaining persistent activation of STAT3 by regulating tumor cells and tumor-infiltrating myeloid cells in primary tumors [12]. S1PR1 and STAT3 stimulate and activate each other to synergistically enhance tumor growth. An interesting study recently identified that S1PR1/STAT3 signaling also induces the recruitment of MDSCs into distant target organs, thereby participating in the formation of the premetastatic niche [13]. It has been confirmed that the S1PR1/STAT3 signaling pathway induces MDSC recruitment in the liver to form a premetastatic niche that promotes colorectal cancer liver metastasis [14]. The exact role of the S1PR1 signaling pathway in lung cancer metastasis still needs further research and exploration.

*Astragalus* is a commonly used tonic herb whose main components, such as *Astragalus* polysaccharide, astragaloside IV (AS-IV), and flavonoids, could effectively stimulate immune function in patients with various diseases. A growing number of studies suggest that APS may exhibit antitumor potential. Our previous findings suggest that APS can affect the autophagy of lung cancer cells, thereby inhibiting the growth of lung cancer. In addition, it can alter the tumor microenvironment to further inhibit lung cancer recurrence and metastasis, as well as have the effect of reducing side effects and enhancing efficacy when used in combination with DDP. However, the mechanism of APS intervention in the occurrence of premetastatic ecotones still needs further study.

## 2. Materials and Methods

### 2.1. Chemicals and Reagents

*Astragalus* polysaccharides (APS) (Yuanye Biological, Shanghai, China) were dissolved physiologically at a concentration of 50, 100, or 200 mg/kg according to the body weight of mice. Fingolimod hydrochloride (FTY720) was obtained from MedChemExpress Co. (New Jersey, USA), dissolved in absolute alcohol, and stored at −20°C.

### 2.2. Cell Culture

Luciferase-labeled Lewis lung cancer cell lines (Luc-Lewis) purchased from Shanghai Fenghui Biology (Shanghai, China) and stored in the ultra-low temperature refrigerator of the Scientific Research and Experiment Center of Gansu University of Chinese Medicine. Take the Luc-Lewis cell line frozen in the laboratory, thaw it rapidly in a 37°C water bath and discard the supernatant after centrifugation (1200 rpm, 5 min). Add 5 mL of complete medium (10% fetal bovine serum (FBS) and antibiotic 1% penicillin streptomycin solution) and then transfer it into a T25 culture bottle. It was cultured in a constant temperature incubator with 5% CO_2_ and 37°C saturated humidity. After the fusion degree of cells reaches about 80%–90%, culture to the required number and digest the cells in the logarithmic growth phase with trypsin to prepare a single-cell suspension. Trypan blue staining detected that the cell activity was greater than 95%; adjusting the number of cells to 1 × 10^6^/*μ*L was used for subsequent experiments.

### 2.3. Animals

There are 90 specific pathogen-free (SPF) C57BL/6J mice, sex in half, weighing (20 ± 2) g, provided by Beijing Forbes Biotechnology Co., Ltd. (License no. SCXK (Beijing) 2019-0010.) The experiment was completed in the animal experiment center of the Gansu University of Chinese Medicine. The experimental conditions are as follows: raised at a specific pathogen-free level, 24 ± 2°C temperature, 50 ± 10% relative humidity, and 12 hours of light/12 hours of the dark cycle. Mice were allowed to adapt to the environment for 1 week before inoculating tumor cells. All experiments were approved by the Institutional Animal Care and Use Committee and were in line with the National Institutes of Health Guidelines for the Use and Care of Laboratory Animals.

### 2.4. Premetastatic Niche Animal Model Construction

Mice were randomly divided into the following groups: a blank group, a model group, an APS low-dose group (APS-L), an APS medium-dose group (APS-M), an APS high-dose group (APS-H) and a Fingolimod group (FTY720), with 15 mice in each group. Except for the blank group, mice in other groups were injected with 1 × 10^6^Luc-Lewis cells via the tail vein for constructing the premetastatic niche model, and the whole process followed an aseptic operation. On the second day of modeling, the model group was given normal saline, and the treatment groups were given the corresponding doses of APS and FTY720, respectively. The mice of the APS-L, APS-M, and APS-H groups were administered intragastrically with aqueous extract at low, medium, and high doses (equivalent to about 50 mg/kg, 100 mg/kg, and 200 mg/kg) once a day. The calculated formula between humans and mice according to the body surface area is mouse do se(g/kg)=human do se(g/kg) × 3/37. The mice in the FTY720 group were injected intraperitoneally with 0.2 mL (1 mg/kg) of FTY720 solution every other day, and the dosing criteria were referred to those in the literature [15]. Until the 28th day, the eyeballs were removed and venous blood was drawn. The test samples were retained after the execution of the mice.

### 2.5. Bioluminescent Imaging

Using an *in vivo* imaging system, we monitored the growth and metastasis of the tumors. Mice were anesthetized with isoflurane, intraperitoneally injected with 15 mg/mL of the XenoLight potassium salt of D-luciferin dissolved in sterile, divalent-free Dulbecco's PBS, and imaged 10 min after the injection. Tumor growth and metastasis were monitored by *in vivo* bioluminescence imaging using the platform of the IVIS spectrum system (IVIS Lumina III, PerkinElmer, USA). The experiments were repeated at least twice.

### 2.6. Hematoxylin-Eosin Staining

On the 14th and 28th days, the lung tissue of each group was collected and kept in 4% paraformaldehyde for 48 h. After gradient dehydration, paraffin was embedded, cut into tissue sections with a thickness of about 5 *µ*m, and placed on glass slides. The sections were stained with hematoxylin and eosin (H&E) staining as per the standard protocol. Histologic changes were observed under a light microscope.

### 2.7. Flow Cytometry

The resected lung was cut into pieces and resuspended with a digestion enzyme mixture (175 U/mL collagenase V, Sigma, GER). The mixture of tissue/enzyme was put in a 37°C shaker for 30 min and then poured through 70 *μ*m cell strainers. Add 2 mL of red blood cell lysate, incubate for 5 min at room temperature, centrifuge, and remove the supernatant. 1 mL of PBS containing 1% FBS was added to obtain a single-cell suspension of the lung tissue. For the detection of MDSCs, FITC anti-mouse/human CD11b antibodies (Biolegend, California, USA) and APC anti-mouse Ly-6G/Ly-6C (Gr-1) antibodies (Biolegend, California, USA) were added. The detection was performed using a FACSCelesta flow cytometer. Data were analyzed using FlowJo software (BD Biosciences, New York, USA).

### 2.8. Western Blot Analysis

Total proteins were extracted from the lung in RIPA buffer with 1 mM PMSF and a phosphatase inhibitor cocktail. Protein quantification was determined by the BCA Protein Assay Kit (Sulaibao, Beijing, China). Samples were equally loaded on polyacrylamide gels and electrophoresed, then electrotransferred to polyvinylidene fluoride membranes and probed with antibodies to S1PR1 (Biorbyt, Cambridge, UK), STAT3 (Abcam, Cambridge, UK), p-STAT3 (Cell Signaling Technology, Massachusetts, USA), and GAPDH (ImmunoWay Biotechnology, Texas, USA). This was followed by incubation with horseradish peroxidase (HRP)-conjugated secondary antibodies. We detect protein bands with enhanced chemiluminescence substrate using a chemiluminescence imager. The signal is quantized by Image *J* software for statistical analysis and processing.

### 2.9. Real-Time PCR

Total RNA was isolated from mouse lung tissues using Trizol reagent, then reverse transcribed into cDNA according to the instructions of the reverse transcription kit, and amplified with a PCR kit (YEASEN, Shanghai, China). Set the amplification procedure as follows: predenaturation at 95°C for 5 min, then denaturation at 95°C for 30 s, annealing at 60°C for 30 s, and denaturation at 72°C for 30 seconds. Repeat 40 cycles in the last 2 steps. The relative expression of RNA was calculated by 2^−ΔΔCT^ methods. The primer sequence is shown in Table 1.

### 2.10. Statistical Analysis

Statistical analyses were performed using SPSS 25.0. All data are presented as the mean ± standard (SD). Student's *t*-test and the two-way ANOVA were used to compare the statistical differences between two groups and between multiple groups, and LSD was used to compare the differences among different groups. *P* values less than 0.05 were considered statistically significant.

## 3. Results and Discussion

### 3.1. Result

#### 3.1.1. APS Decoction Inhibited the Formation of Lung Premetastatic Niche

**Figure 1:**
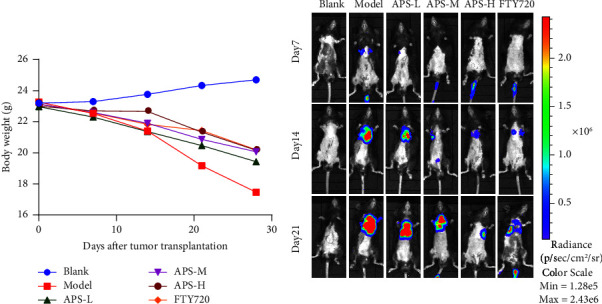
APS inhibited the formation of the lung premetastatic niche and thus prevented metastasis. (a) Changes in body weight in the blank group, model group, APS-L group, APS-M group, APS-H group, and FTY720 group over 28 days. (b) The small animal live fluorescence imaging in the blank group, model group, APS-L group, APS-M group, APS-H group, and FTY720 group on days 7, 14, and 21 after tumor inoculation.

The changes in the body weight of mice were recorded daily. Compared with the blank group, the weight of mice in the model group decreased significantly, and the weight of mice in the treatment group also showed some degree of weight loss, accompanied by obvious shortness of breath, listlessness, decreased mobility, and poor fur luster. There was no significant difference in body weight between the APS-H group, the FTY720 group, and the blank group. As shown in Figure 1(a), on the 7th, 14th, and 21st days after inoculation, tumor fluorescence signals in mice were dynamically monitored by bioluminescence. On day 7, except for the blank group, faint fluorescent signals could be observed in the lungs of the model group and the APS-L group; in the remaining groups, fluorescence expression was only observed in the tail. On the 14th day, the tumor fluorescence expression was enhanced in the lungs of mice in the model group, and weak fluorescence signals could be detected in the lungs of other groups. On day 21, the fluorescence signal expression of tumor cells in the model group was significantly enhanced, and metastasis to other organs occurred. Compared with the model group, the fluorescence expression of each treatment group was decreased to varying degrees. As shown in Figure 1(b), we found that the fluorescence signal was mainly concentrated in the two lung regions with strong light spots, and the expression of photon number in the right lung was significantly stronger than that in the left lung, which was consistent with the characteristics of tumor cell hematogenous dissemination. Taken together, according to the results of the experiment, the period of the premetastatic lung niche was approximately set at 13–15 days. The APS treatment group and the FTY720 group have different degrees of inhibition on lung metastasis, among which the APS-H group and the FTY720 group have the most obvious inhibition.

#### 3.1.2. Histopathological Changes of the Lung in Mice

**Figure 2:**
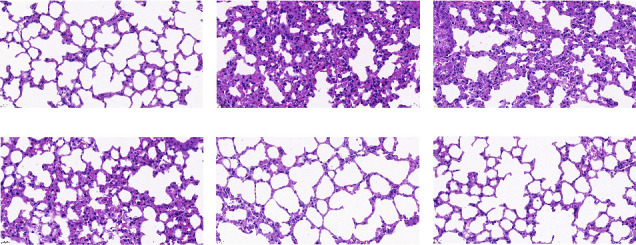
The morphologic changes in the lung tissue of mice on day 14. (a) Blank group. (b) Model group. (c) APS-L group. (d) APS-M group. (e) APS-H group. (f) FTY720 group (magnification of ×400). The data were presented as the mean ± SD. *n* = 6. Scale bar = 20 *μ*m.

**Figure 3:**
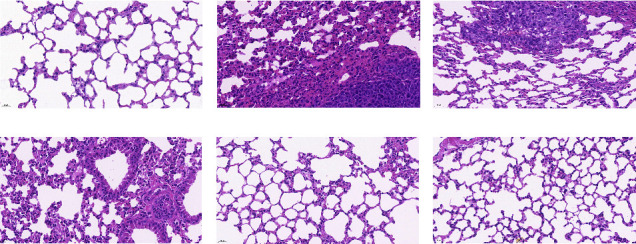
The morphologic changes in the lung tissue of mice on day 21. (a) Blank group. (b) Model group. (c) APS-L group. (d) APS-M group. (e) APS-H group. (f) FTY720 group (magnification of ×400). The data were presented as the mean ± SD. *n* = 6. Scale bar = 20 *μ*m.

The results of HE staining showed that the blank group had normal structures of the bronchioles, alveolar wall, and alveolar epithelium, a thin alveolar septum, an intact and uniformly sized alveolar lumen, and no exudate or inflammatory cell infiltration. On the 14th day after inoculation, compared with the blank group, the model group had thickened septa, disrupted alveolar structures, and infiltrated inflammatory cells. Compared with the model group, the lung tissue morphology of each treatment group showed different degrees of improvement, while there was no significant difference between the APS-H group, the FTY720 group, and the blank group, as shown in Figure 2. On the 21st day, compared with the blank group, the tumor cells with large and hyperchromatic nuclei gathered significantly in the model group, and disordered cancer nests were formed. The surrounding fibrous tissue was disrupted and heavily infiltrated by inflammatory cells. Compared with the model group, the tumor cells in the treatment group were less aggregated, and the lung tissue structure was basically clear in a dose-dependent manner. Among them, the APS-H group and the FTY720 group had significant therapeutic effects, as shown in Figure 3.

#### 3.1.3. APS Inhibited the Content of MDSC in the Lung

**Figure 4:**
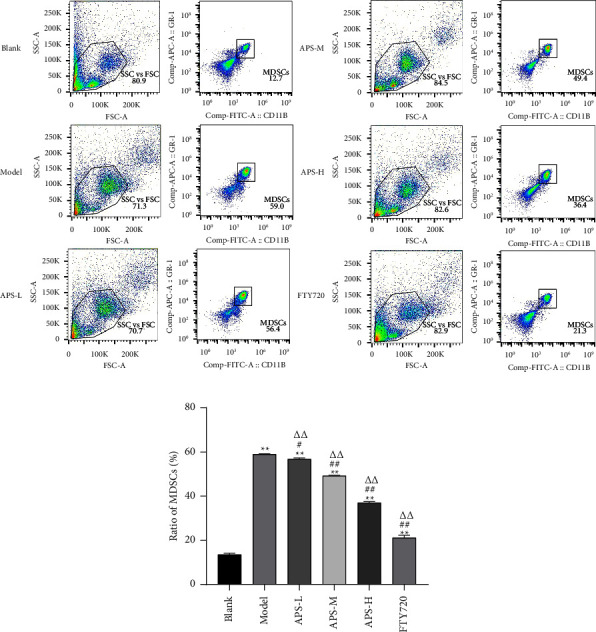
APS inhibits the content of MDSCs in the lung. (a) Flow cytometry detected the number of MDSCs in the lung. (b) The histogram showing the content of MDSCs in the lungs of each group. The data were presented as the mean ± SD. *n* = 6. ^*∗∗*^*P* < 0.01, compared with the blank group; ^#^*P* < 0.05, ^##^*P* < 0.01, compared with the model group; ^ΔΔ^*P* < 0.01, compared with the FTY720 group. Control by one-way analysis of variance (ANOVA) with LSD.

We detected the percentage of MDSCs in the lungs of mice by flow cytometry. As shown in Figure 4, on day 14 after implantation of tumor cells, the results showed that the percentage of MDSC significantly increased in the lung of the model group compared with the blank group. The MDSCs in the lungs of mice decreased after APS treatment, with the most significant decrease in the percentage of MDSCs in the APS-H and FTY720 groups (*P* < 0.05). Remarkably, APS decreased the content of MDSC in the lungs.

#### 3.1.4. APS Inhibited the Expression of S1PR1/STAT3 Pathway-Related Molecules in Premetastatic Niche of the Lung

**Figure 5:**
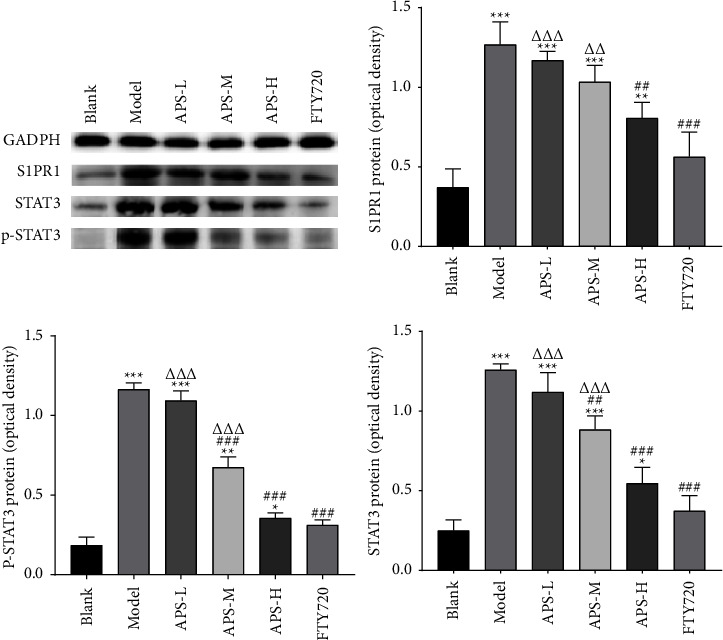
Western blot analysis for the expressions of S1PR1, STAT3, and p-STAT3 in the blank group, model group, APS-L group, APS-M group, APS-H group, and FTY720 group. The graph represents the relative densitometric intensity of each band normalized to GAPDH. The data were presented as the mean ± SD. *n* = 6. ^*∗*^*P* < 0.05, ^*∗∗*^*P* < 0.01, and ^*∗∗∗*^*P* < 0.001, compared with the blank group; ^##^*P* < 0.01, and ^###^*P* < 0.001, compared with the model group; ^ΔΔ^*P* < 0.01, compared with the FTY720 group. Control by one-way analysis of variance (ANOVA) with LSD.

**Figure 6:**
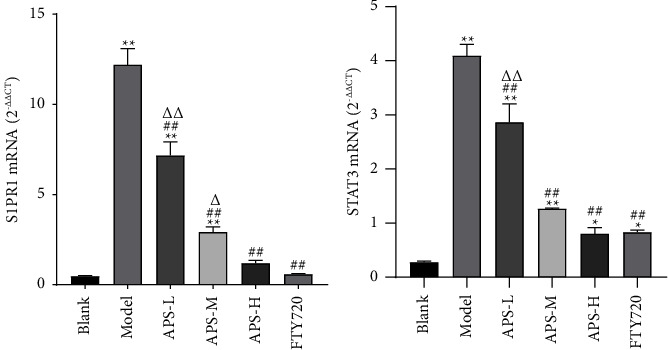
The expression levels of the S1PR1 and STAT3 mRNA in different groups. These results are consistent with the trend of protein expression. These results are consistent with the trend of protein expression. These data were calculated according to 2^−ΔΔCT^ with the blank group as the control group. The data were presented as the mean ± SD. *n* = 6. ^*∗*^*P* < 0.05 and ^*∗∗*^*P* < 0.01, compared with the blank group; ^##^*P* < 0.01, compared with the model group; ^Δ^*P* < 0.05 and ^ΔΔ^*P* < 0.01, compared with the FTY720 group. Control by one-way analysis of variance (ANOVA) with LSD.

To further explore the effects and molecular mechanisms of improvement of the premetastatic niche of the APS, as shown in Figure 5, Figure 6,and Table 2, we employed Western blotting and qRT-PCR to detect the S1PR1/STAT3 pathway-related proteins and genes. We observed that S1PR1, p-STAT3, and STAT3 expression in the model group was markedly higher than that in the blank group (*P* < 0.05); compared with the model group, the relative expression of S1PR1, p-STAT3, and STAT3 in the lung tissue of mice in APS treatment groups and FTY720 groups decreased in a dose-dependent manner (*P* < 0.05). There was no significant difference in the expression between the APS-H group and the FTY720 group (*P* > 0.05). Taken together, these results demonstrated that APS regulated the S1PR1/STAT3 pathway, which contributed to the effect on MDSC.

## 4. Discussion

Clinical data show that tumor metastasis is the main cause of death in cancer patients [16]. Although surgical intervention, immunization, and targeted therapy have been applied in clinic, the tumor metastasis rate and postoperative recurrence rate are still high. In 2005, Kaplan et al. [17] first proposed the concept of “the premetastatic niche” based on the “seed and soil theory,” which states that before the tumor cell metastatic dissemination, the primary tumor can secrete factors that contribute to the generation of PMN. This niche is distant from the primary tumor, which is suitable for the outgrowth and colonization of incoming circulating tumor cells. Its formation requires the interaction of three major factors: primary tumor-derived molecular components; regulatory and suppressive immune cells; and the proinflammatory polarization of the local stromal microenvironment of the host (or future metastatic organ components). Recent studies have shown that PMN plays an indispensable role in the process of tumor metastasis to the lung [18, 19]. However, the complex process and molecular mechanism of PMN formation remain one of the greatest mysteries surrounding cancer metastasis.

Traditional Chinese medicine, with its high efficiency and multitarget characteristics, has played a unique role in the treatment of cancer patients, so it may be regarded as a potentially effective strategy for the treatment of cancer [20]. According to the theory, “Preventing a disease before it arises” is not only an important theory of traditional Chinese medicine but also one of its important guiding ideologies in treating tumors and preventing metastasis. That is, when treating tumors, we should first protect the undamaged areas and emphasize the combination of enhancing body resistance and eliminating pathogenic factors. *Astragalus membranaceus* is a commonly used traditional Chinese medicine to tonify the qi of the spleen and stomach. Its extract, APS, has many pharmacological effects, such as regulating body immunity, enhancing macrophage activity, having an antitumor effect, being anti-inflammatory, and so on [21]. Studies have shown that APS can be used as an effective modulator of tumoral M1/M2 macrophage polarization and a potent activator of DC maturation so as to play an immune-dependent anticancer role in lung cancer [22]. In gastric cancer cells, APS can not only promote GC cell apoptosis and decreases the viability through the AMPK pathway, but also enhance the antigastric cancer effect of Adriamycin may as a chemosensitizer [23]. It can also significantly decrease the migration and invasion of breast cancer cells *in vitro* by inhibiting EMT and modulating the expression of components of the Wnt/*β*-catenin signaling pathway [24]. According to the therapeutic principle of “Reinforce healthy qi to strengthen the body,” we explored the effect of APS on the S1PR1/STAT3 signaling pathway and the intervention mechanism on PMN. S1P receptor modulator FTY720, due to its resemblance to sphingosine in chemical structure, fingolimod can act as a sphingosine kinase substrate, and it is phosphorylated by these kinases and turned into active fingolimod-P, which competitively binds to S1PR1 on the cell membrane surface *in vivo,* thereby inhibiting the action of S1P and exerting immunosuppressive and immunomodulatory functions, and thus exerting anticancer effects. It has been demonstrated that FTY720 blocks the SPHK1/S1P/S1PR1 axis, leading to the blockade of the NF-kB/IL-6/STAT3 amplification loop and colitis-associated cancer [25]. In a recent study, it was demonstrated that FTY720, by suppressing the S1PR1/STAT3 loop, inhibited tumor growth and desmoplasia and suppressed resistance to the chemotherapy drug gemcitabine [26].

By constructing a premetastatic niche in a lung model and using bioluminescent imaging and HE staining techniques, we determined the formation time of the premetastatic niche more truly and intuitively. The results showed that on the 14th day, except for the blank group, the fluorescence signals of tumor cells could be observed in the lung tissues of other groups of mice, and the intensity of fluorescence expression was positively correlated with the modeling time. On the 21st day, the fluorescence expression in the lung was significantly enhanced, we speculated that the premetastatic niche might mature on the 14th day, and the metastatic lesion had been formed on the 21st day. We observed that, compared with the blank group, the lung tissue of the model group mouse on the 14th day was a deep red color, with thickened lung septum, alveolar atrophy, and inflammatory cell infiltration. On the 21st day, metastatic nodules were seen in the lung of the model group, with large and hyperchromatic tumor cell nuclei seen in pathological sections. Therefore, if the abovementioned speculation is verified, it was determined that 13–15 days after inoculation was the mature stage of the premetastatic niche. There are similarities to the results that have been published [17]. In this study, we dynamically monitored the metastatic pathways of tumor cells by biological imaging and observed the pathomorphology of the lung tissue by HE staining, and found that APS improved the quality of life in mice, protected the structural integrity of lung tissue, improved inflammatory infiltration, and delayed the progression of tumor lung metastasis. Studies have shown that tumor progression is related to MDSCs in the tumor microenvironment, and the increase in the number of MDSCs is related to poor prognosis [27]. MDSCs represent the heterogeneous population of immature myeloid cells, which are generated in the bone marrow and finally recruited in the premetastatic niche of the lung, thus inducing the characteristics of local immunosuppression, while tumor cells use the characteristics of immune mechanisms to skillfully escape the recognition of the body's immune system, survive, and proliferate in the body, supporting disease progression [28, 29]. In this study, after APS treatment, we found that the proportion of MDSCs in lung tissue decreased in a dose-dependent manner by flow cytometry. This suggested that APS may inhibit MDSCs' recruitment into the lungs, where the premetastatic niche is set up. In normal cells, the activation of STAT3 transmits the transcriptional signals of cytokines and growth factor receptors to the nucleus, generally by phosphorylation [30]. In contrast, in most cancer processes, hyperactivity of STAT3 is commonly correlated with a poor clinical prognosis [31]. Continuously activated STAT3 in tumor cells as a pivotal oncogenic mediator and an effective transcription factor has been extensively studied [32]. S1PR1 activation is the key factor for the continuous expression of STAT3 protein in the premetastatic niche, and it is also the premise for the microenvironment to gradually mature [33]. It has become the research highlight of the world to achieve the purpose of antitumor metastasis by interfering with the S1PR1-related signal pathway [34]. The activation of the S1PR1/STAT3 signaling pathway enables BMDCs to cross blood vessels and mediate the continuous survival and proliferation of themselves and other stromal cells [35]. There is a positive feedback loop between S1PR1 and STAT3. S1PR1 is a factor leading to the sustained activation of STAT3. STAT3 is the transcription factor most related to the amplification of MDSCs, which induces the expression of S1PR1 [11]. Therefore, the continuous activation of STAT3 can inhibit the differentiation of BMDCs into mature myeloid cells and then promote the expansion of MDSCs. Of note, through the results of WB and RT-PCR, we found that APS can reduce the protein and gene expression of S1PR1, STAT3, and p-STAT3 to play a therapeutic role. The above results suggest that APS can, via the S1PR1/STAT3 signaling pathway, inhibit MDSC recruitment into the lung so as to interfere with the formation of the premetastatic niche.

## 5. Conclusions

The present study suggested that APS can reduce the expression of S1PR1 and downstream molecules STAT3 and p-STAT3 and inhibit the accumulation of myeloid-derived suppressor cells in the premetastatic niche of the lung via the S1PR1/STAT3 pathway, thus indicating that APS may be considered as a potentially effective strategy for the treatment of patients with lung cancer.

## Figures and Tables

**Table 1:** Sequences for RT-qPCR primers.

Names	Primer sequence	Product (bp)
S1PR1	Forward: 5′-GTGTTCATTCTCATCTGCTGCTTC-3′	225
	Reverse: 5′-AAACATACTCCCTTCCCGCA-3′	

STAT3	Forward: 5′-TGCGGAGAAGCATTGTGAGTG-3′	210
	Reverse: 5′-TCTTAATTTGTTGGCGGGTCT-3′	

GAPDH	Forward: 5′-CCTCGTCCCGTAGACAAAATG-3′	133
	Reverse: 5′-TGAGGTCAATGAAGGGGTCGT-3′	

**Table 2:** The expression levels of S1PR1 and STAT3 mRNA in different groups (mean ± SD; *n* = 6).

Groups	S1PR1	STAT3
Blank	0.47 ± 0.03	0.28 ± 0.02
Model	12.2 ± 0.89^*∗∗*^	4.09 ± 0.21^*∗∗*^
APS-L	7.17 ± 0.76^##^^*∗∗*^^△△^	0.34 ± 0.02^##^^*∗∗*^^△△^
APS-M	2.92 ± 0.29^##^^*∗∗*^^△^	1.26 ± 0.02^##^^*∗∗*^
APS-H	1.19 ± 0.16^##^	0.08 ± 0.11^##^^*∗*^
FTY720	0.59 ± 0.03^##^	0.83 ± 0.04^##^^*∗*^

^*∗*^ *P* < 0.05 and ^*∗∗*^*P* < 0.01 vs. the blank group; ^##^*P* < 0.01 vs. the model group; ^Δ^*P* < 0.05, ^ΔΔ^*P* < 0.01 vs. the FTY720 group.

## Data Availability

The data used to support the findings of this study are available from the corresponding author upon reasonable request.
